# “It cannot be boring!”: Developing a measure of function for young adults accessing integrated youth services

**DOI:** 10.1186/s41687-022-00491-6

**Published:** 2022-09-03

**Authors:** Skye Barbic, Emily Brooks, Natalia Lassak, Mo Khaleghi, Marco Zenone, Nikki Ow, Adelena Leon, Steve Mathias

**Affiliations:** 1 Department of Occupational Science and Occupational Therapy, The University of British Columbia, 2211 Wesbrook Mall T325, Vancouver, BC V6T 2B5 Canada; 2 Foundry, 915-1045 Howe Street, Vancouver, BC V6Z 2A9 Canada; 3 Providence Research, 10-1190 Hornby Street, Vancouver, BC V6Z 2K5 Canada; 4 Centre for Health Evaluation Outcome Sciences, 588-1081 Burrard Street, Vancouver, BC V6Z 1Y6 Canada; 5 Department of Psychiatry, The University of British Columbia, 2255 Wesbrook Mall, Vancouver, BC V6T 2H1 Canada

**Keywords:** Function, Mental health, Substance use, Youth, Young adults

## Abstract

**Background:**

In Canada, the prevalence of mental health challenges is highest in young people aged 12–24. Mental health challenges frequently cause marked functional impairment. Despite this, we are unaware of any existing conceptualization and/or measures of *function* that has been developed from the perspective of young people. The objective of this paper is to develop a conceptual and measurement model, including a preliminary set of items, for an outcome measure of function for young adults accessing mental health services.

**Methods:**

We conducted this study in three phases. In phase 1, we conducted three focus groups to conceptualize function as a construct from the perspective of young adults. In phase 2, we co-designed a set of items with youth (n = 4) to capture the construct. In phase 3, we invited young people (n = 12) accessing mental health services to complete workbooks and participate in one of two focus groups to evaluate whether items were clear, captured function comprehensively, and were relevant. We transcribed and compiled all data to eliminate, refine and generate new items.

**Results:**

In phase 1, a conceptual model of function was developed with three main themes: basic needs, roles and responsibilities, and social connections**.** In phase 2, 97 candidate items were developed, and in phase 3, a candidate pool of 50 items resulted for psychometric testing.

**Conclusion:**

This youth-centred conceptualization of function and preliminary item bank has the potential to advance person-centred care, outcomes, and experiences for youth seeking mental health services.

**Supplementary Information:**

The online version contains supplementary material available at 10.1186/s41687-022-00491-6.

## Introduction

An estimated 1 in 5 Canadians experience mental health issues annually, with the highest prevalence in those aged 15–25 [1]. Mental illness costs the Canadian healthcare system upwards of $51.1 billion in direct healthcare services, excluding peripheral costs associated with caregiving and lost employment [2]. In response, British Columbia has responded by developing an integrated primary model of care to support the needs of youth (12–18 years), young adults (19–25 years), and their families/caregivers. The service model is called Foundry (foundrybc.ca). Since 2018, Foundry has provided integrated primary care (including sexual health), mental health, substance use, peer support, and social services (i.e., work/study support) to nearly 40,000 young people. Currently, twelve Foundry centres exist in British Columbia, with eleven more in development [3].

Early learnings at Foundry highlight that ‘function’ is an important outcome for young people, their families, service providers, and funders [3–6]. Function, including physical, psychological, social, and occupational function, has been shown to have a significant association with health (mortality and morbidity) and social outcomes (i.e., social relationships, living circumstances, education and employment [4, 5, 7, 8]. Youth and young adults with mental health challenges frequently face barriers to function, including barriers to education/employment, safe and affordable housing, and timely access to health services, [9–11]. They also face barriers to communicating function to health providers, including a lack of youth-centred measures [4, 5], lack of co-designed measures that are culturally relevant [12], and measures that create a common language for guiding care [13]. “Function”, as it is currently conceptualized and measured, is often associated with mental well-being and/or quality of life [14–20]. However, to measure function from the perspective of youth and young adults, a hypothesized conceptual framework and youth-centred language of outcomes is needed. As Patrick and colleagues [21] note in their landmark paper *Patient-Reported Outcomes to Support Medical Product Labeling Claims: FDA Perspective*, we must “Begin with the End in Mind” (p.S127). The goal of this project is to develop a youth-centred model and measure of function that enable treatment plans that are fit for purpose for this population and driven by what is meaningful to diverse youth and young adults [22].

The universal model of function, the International Classification of Function (ICF) defines the term as “all bodily functions, activities and participation,” and conceptualizes these facets as interacting with contextual and individual factors (World Health Organization [WHO] [23], p. 2). Since the model depicts all activities and participation as equally important to function, it is not surprising that current measures of function measure varying constructs. However, existing measures of function are predominantly clinician-reported, including the Global Assessment of Functioning (GAF), the Personal and Social Performance Scale (PSP), the Social and Occupational Functioning Assessment Scale (SOFAS), and the Life Functioning Assessment Inventory (L-FAI) [24–28]. Patient-reported measures that do exist [(i.e., WHODAS, Kessler Distress Scale (K10)] focus on general domains of function relevant to the adult population or are designed to capture epidemiological trends rather than designed to evaluate the effectiveness of interventions over time [29–31]. The substantial gap between the outcomes captured in research and in youth-centred health care are increasingly shown to be detrimental for youth, providers, clinics, and health care systems. At this time, there is a need for a measure of function that can be used to evaluate the effectiveness of integrated youth services and to inform quality improvement initiatives [32]. It is also paramount that a new measure considers the modern day priorities of youth, including considerations for equity and diversity, co-deign methods, and including those who may have barriers to accessing services in the first place [12, 33].

Patient Reported Outcome Measures (PROM’s) are one type of assessment tool that can help young people communicate concerns to their healthcare providers and monitor their own progress [34]. By bolstering young peoples’ self-efficacy through meaningful measurement, assessments of function can empower young adults and families to engage in care that is contextualized based on their needs and priorities [35]. The objective of this study was to develop an initial item set to capture the concept of “Function” from the perspective of young people accessing integrated health services.

## Methods

**Fig. 1:**
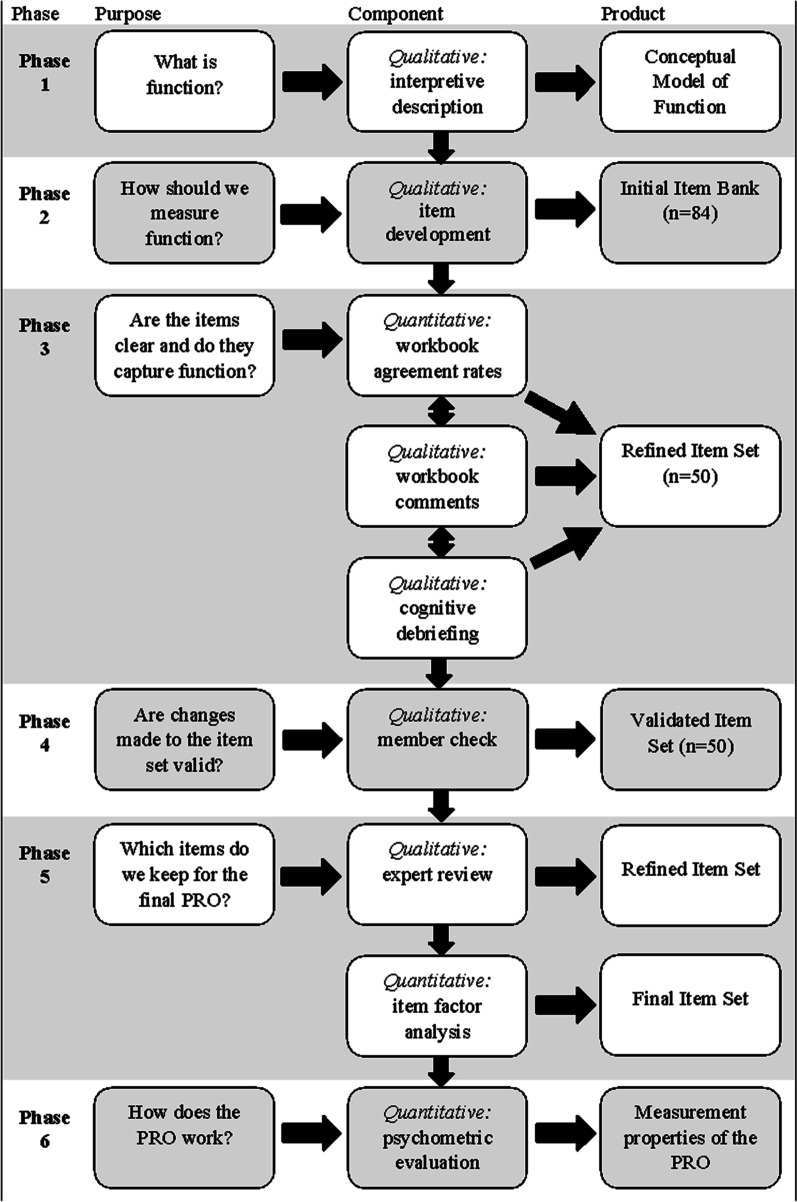
Mixed-methods patient-reported outcome (PRO) development process

This study describes the first three phases of measurement development as depicted in Fig. 1.

### Conceptualization of function (Phase 1)

We used a qualitative study design guided by focus-group methodology to answer our research questions. Before beginning, we hired a youth research lead (MK) and a team of youth research assistants to co-design the interview schedule and to suggest opportunities to maximize opportunities for diverse youth to participate in the project. The team co-designed the questions (see Additional file 1: Table S1). Using posters, we championed the project and recruited young people from an integrated youth service network called Foundry (foundrybc.ca) that provides health and wellness services and support to youth and young adults with mental health and substance use disorders in British Columbia, Canada. For this study, we recruited young people from one centre located in Vancouver, an urban centre, between November and February 2018/2019, with the goal of first identifying function from the perspectives of young adults who experience mental health and/or substance use disorders. Participant inclusion criteria was young people between 19 and 25 years of age diagnosed with a mental health and/or substance use disorder, willingness and ability to read and respond in English, and provide informed consent. Based on youth engagement and a review of the literature, it was felt to be important to focus uniquely on young adults separately before youth, as their developmental needs were different (Kwan et al. [36]). Prior to study enrollment, participants were explained the study purpose, expectations of them as a participant, and given contact information of the lead author and University of British Columbia (UBC) research ethics board.

Our team, including two trained youth research assistants and the primary investigator, conducted two 90-min focus groups, with a plan for more if saturation was not achieved. We aimed to have 5–9 participants in each focus group to allow for meaningful participation of each young person and to account for the new experience of youth with this methods. Participants were invited to one focus group. We recorded and manually transcribed the focus groups verbatim. Two team members and two participants (one for each focus group), reviewed the transcripts. Subsequently, two researchers not present for the focus groups, analyzed transcripts to code for themes, employing an inductive thematic approach to identify themes representative of function in young adults. To assess preliminary evidence of saturation, we reviewed each focus group discussion in order in which groups were conducted and documented the development of codes. Specifically, we documented all new codes that were developed, their characteristics, including the code definitions, type of code, notes about rationale for new codes (youth-centred, clarity of the code, completeness of the code), and whether any previously developed codes were present in the transcripts. We also took notes on the evolution of the coding, recording changes made to the codes and rationales where appropriate. As outlined by Korstjens and Moser [37]’s practical guide, we also implemented several trustworthiness strategies, including triangulation and member checks with five randomly selected participants.

For triangulation, three researchers interpreted the data independently, after which we compared interpretations. Where there were differences, we came together as a team to discuss them until the most suitable interpretation was found that represented the data (Korstjens and Moser). The researchers held weekly meetings during the analytical process and we held monthly analysis sessions with the research team. For member checking, halfway through (n = 3) and at the end of the study period (n = 2), we went back to participants for feedback and asked them to review the interpretation of the data and challenge what they perceived as correct or in accurate interpretation of the data. This iterative process continued until the codebook was complete and our team agreed that the trustworthiness of the data was excellent. We developed an understanding of the broad themes that constitute function in young adults by (1) analyzing the data independently before comparing respective codes and co-constructing the overarching themes through an iterative process of ongoing discussion (2) reflecting on our positionality as researchers, and (3) incorporating varying participant descriptions of function with written reflections from the focus group interviewers to build successively more sophisticated reconstructions. This process was modelled on the work of Klassen and colleagues [38].

### Item development (Phase 2)

Our team (including one youth researcher, a young adult with lived experience of mental health challenges and who has accessed integrated youth services) developed items based on nouns and verbs collected from the phase 1 transcripts and conceptual model. As a team, we endeavored to develop at least one item for each theme identified to ensure the item bank covered the full breadth and range of function in young adults, from low to high. As outlined by Thrush [39], we used plain English to increase transferability of the item bank’s language to different social and geographical contexts and increase accessibility to non-native English speakers. However, we also judiciously retained the word choices used by participants in our prior research when appropriate, to better capture participants' original sentiments. At this point, we also designed the instructions for completing the tool and proposed category response scales.

### Cognitive debriefing (Phase 3)

We held two focus groups at a conference room in a central location accessible to young people. Participants were recruited purposefully from a local integrated youth service. The youth peer researcher (MK) was available to any young person to ask questions about the study. The youth peer researcher also supported screening of young people to ensure they met the criteria for participating in this phase (age 19–25, accessing integrated youth services, and have current/past experience with mental health challenges) and that our sample for this phase was diverse and representative of young adults accessing this health centre, including youth who were precariously housed, racialized, and/or gender-diverse.

**Fig. 2:**
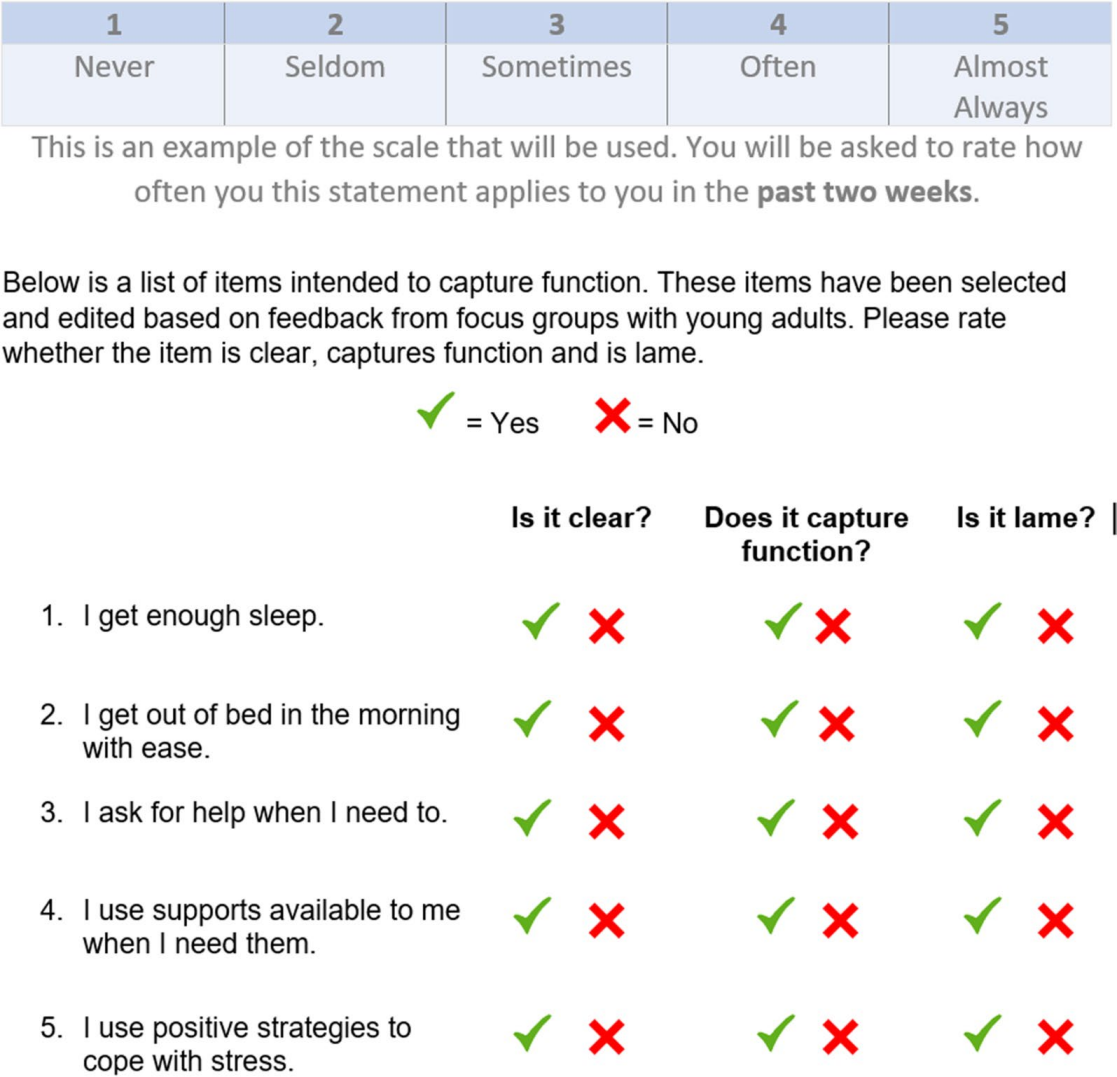
Example of structure to test items in Phase 3

At the start of each focus group, participants individually completed a workbook containing all candidate items from Phase 2 (n = 84). Without conferring, we asked participants to circle yes/no in response to two questions: From your perspective, (1) “is it [the item] clear?” and (2) “does it [the item] capture function and (sub-question) does it capture function comprehensively? This generated quantitative data for researchers to calculate concordance rates between participants from both focus groups. Qualitative feedback and input about item comprehensive was also collected and reported back to each group. Based on feedback from the first focus group participants, a third category was added to the workbook to assess whether each item was youth friendly or meaningful. To preserve original phrasing and the wishes of our participants, we added a third question: (3) “is it [the item] lame?”. Additionally, space was provided below each item for participants to provide more detailed feedback or alternate wording suggestions or apparent missing items to capture comprehensiveness. Of note, the use of the term “lame” was discussed at length throughout this. Our team returned to participants several times to clarify if changing the term to “boring” was sufficient to capture the intent of participants, not so to promote ableist language. Participants accepted the change, but emphasized that *“items cannot be lame/boring or youth won’t buy into the process of filling in measures”.* See Fig. 2 for an example of how this was worded in the participant workbook.

Participants subsequently expressed their interpretations of each item in a focus group discussion. Our team used verbal prompts to explore varying interpretations of each item and promote equitable participation. Prompts included, “what does this item mean to you?” and, “can you elaborate on any confusion you had in understanding this item?” based on recommendations from Patrick et al. [21]. At the end of the focus groups, we asked participants whether the items any content salient in capturing function from the perspectives of young adults. Two sources of data included the workbooks and the focus group data.

#### Workbook data and focus group data analysis

Our team recorded and transcribed the focus groups verbatim. Subsequently, two researchers coded the focus groups separately to ensure the credibility of the findings, which is pertinent in the development of patient-reported outcome measures [21]. As outlined by Korstjens and Moser [37]’s practical guide, our team implemented several trustworthiness strategies, including method triangulation and investigator triangulation. We triangulated the concordance rates from the workbook with the comments from the focus groups. The participants completed the workbook prior to the focus group, enabling them to share their initial impressions of the item in private and without external influence. In the subsequent focus group, participants explored their ideas in more depth and elaborated on responses that needed clarification [40]. Second, our team triangulated findings by separately coding focus group transcriptions, analyzing the findings, deciding which items to keep, modify, or eliminate, prior to corroborating their findings [37].

We pre-determined that any item to which less than 70% of participants answered “yes” to the workbook questions “is it clear?” and “does it capture function?” would be eliminated. We also eliminated any item where more than 70% of participants answered yes to the question “is it lame?”. We used qualitative data from the focus groups to eliminate or refine items that fell above/below the cut-off rates. We retained items that could not be agreed upon for member phases of measurement development.

## Results

### Conceptualization of function (Phase 1)

#### Demographics

Nineteen participants made up two focus groups. Participants ranged from 19 to 25 years old, with a median age of 24 years old. Seven (37%) participants identified as female, twelve (63%) identified as male and one participant (5%) identified as non-binary. The majority of participants (63%) identified as White. Six (32%) identified as First Nations/Metis/Inuit, three each identified as South Asian, Black/African and Hispanic/Latino (16%), and two identified as Middle Eastern/North African (11%). Two participants (11%) were attending school only, three (16%) were employed, one (5%) was attending school and had employment, while twelve (63%) participants were unemployed and not in school. Seven (37%) participants were looking for employment, and six (32%) were not. Six (32%) participants had a high school diploma and seven (37%) had some high school. Three (16%) participants had some college or technical school education, two (11%) had some university education, and one (5%) had a bachelor’s degree. Ten (53%) reported living in a single room occupancy (SRO) hotel, group home, or Covenant House. SROs are a low-cost housing option in Vancouver typically made up of single rooms ranging from 8-12m^2^ in size, including a sink, hot plate, and shared washroom facilities [4, 5]. Four (21%) participants were living with someone else and six (32%) were living in an apartment. Participants’ self-reported mental health diagnoses included mood disorders (79%), anxiety disorders (74%), post-traumatic stress disorder (PTSD) (32%), other disorders (32%) and psychotic disorders (16%). Most participants (69%) reported using alcohol, 53% reported using cannabis, and 31% reported using substances in the last two weeks.

#### Conceptual model

**Fig. 3:**
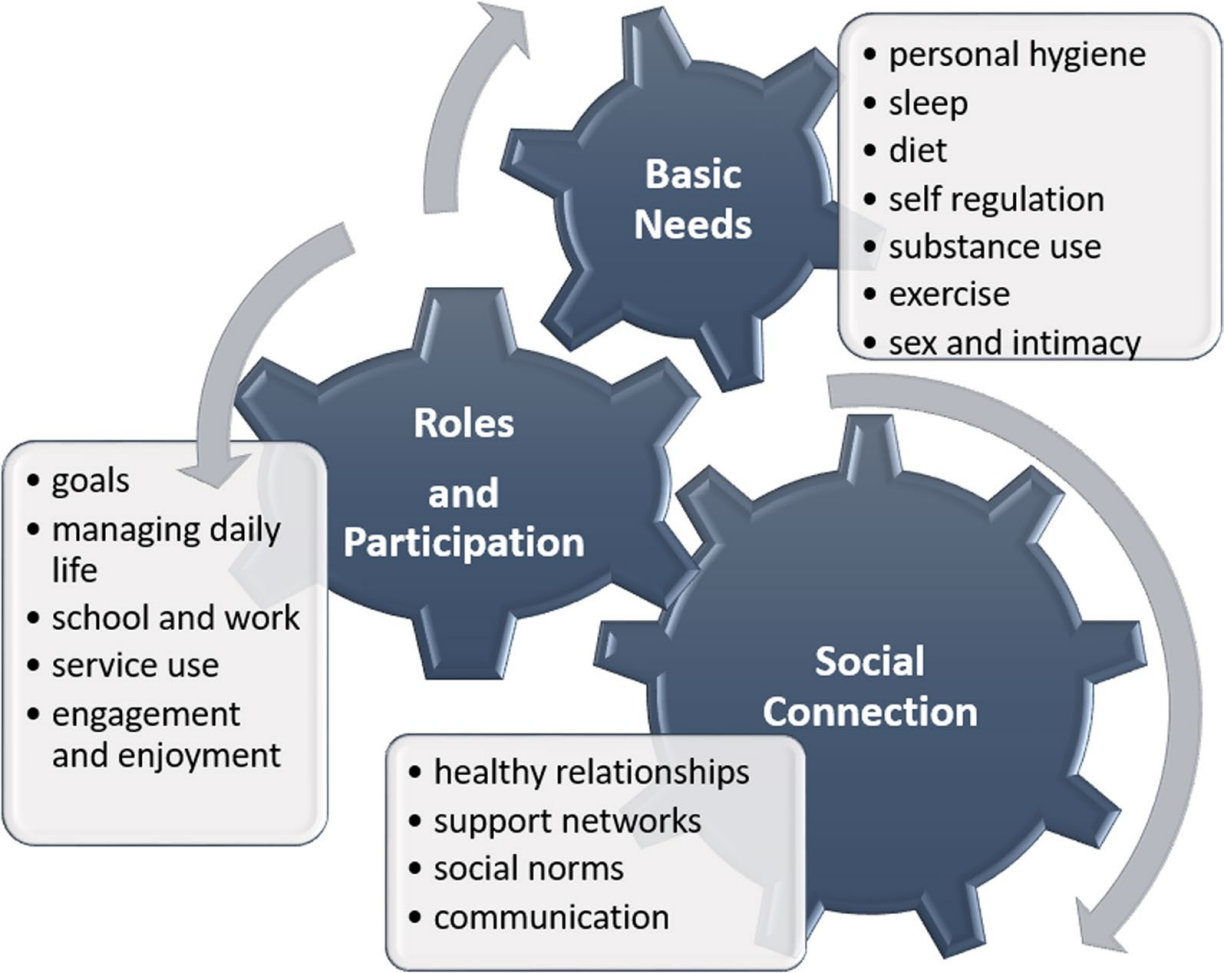
Depiction of conceptualization of function from the perspective of young adults with mental health challenges accessing integrated youth health services in an urban setting. The model has three integrated parts: (1) basic needs, (2) roles and participation, and (3) social connection

Participants provided diverse and wide-ranging definitions of function, which fit into three broad themes of (1) basic needs (2) roles and participation and (3) social connection described in Fig. 3. Basic needs include the subcategories: diet, sex, self-regulation, substance use, personal hygiene, sleep and exercise. Roles and participation include the subcategories of school and work, exercise, goals, engagement and enjoyment, service use and managing daily life. Finally, social connection includes the subcategories of healthy relationships, communication, social norms and support networks.

#### Spectrum of function

Participants described a spectrum of function, from low to high. Additionally, participants depicted various elements, such as self-regulation, participation and social relationships as interacting, leading to downward spirals. One participant described, *“if lots of things are not going the right way in your life, you might be stressed which would lead to more low function.”* Conversely, participants described how elements can snowball and build functional gains, describing *“let’s say you finish part of your studies, or you find something that was important to you… I feel it gives you the confidence and inspires the drive to pursue more or go further down the path.”* Numerous other elements were described as interrelating; components of managing daily life, such as organizing time, were depicted as highly related to basic needs such as diet or sleep, with participants describing the importance of *“eating habits and sleep routines.”* Participants framed goals relative to *“societal productivity standards,”* and *“milestones”* therefore sharing a close relationship with social norms and *“fitting in.”* Additionally, one participant noted how healthy relationships shape self regulation, stating that when “*surrounded by people who have confidence in you, you have confidence in yourself.”* Given this, our conceptual model depicts these themes as dynamically interacting (Fig. 3).

#### Basic needs

##### Sleep

Participants highlighted the importance of a sleep schedule, and expressed that low function involved “*not sleeping for 3 days straight.”* In addition to getting enough sleep, participants noted that during periods of higher function they were “*able to just get up.”*

##### Self-regulation

Participants spoke of how function well involves actively controlling thoughts, emotions, attention and focus. Participants shared that function entails “*establishing and maintaining emotional stability,”* “*being aware of your thought patterns”* and having *“control of your thoughts.”* Participants highlighted that controlling your thoughts might entail *“different coping skills for when the situation occurs.”* Other self-regulation strategies include *“building exposure to your triggers”*, *“mindfulness”*, being *“aware of self-harm”* and *“processing things, in words of, like, how an operation or situation goes.”*

##### Safe substance use

One participant said low function usually entails *“trying to sedate myself with drugs.”* Others suggested higher function entails “*controlling your substance use.”* Substance use was not restricted to elicit substances, with participants noting it *“could be from cigarettes or hard drugs.”*

##### Personal hygiene

Participants described that function well entails one to “*brush your teeth and maintain proper hygiene”,* while the lower end of function involved *“not partaking in personal hygiene habits.”*

##### Exercise

Participants noted that exercise is a central component of health that is integral to function. One participant described that during high function periods they *“move daily.”*

##### Diet

Participants highlighted that function well entails “*eating right”;* however, on the lower end of the spectrum, *“you don’t keep good eating habits.”*

##### Sex and intimacy

Participants also expressed that having safe sex is integral to function. As one participant noted, “This is important S@$* that not one talks to us about, but man it is all I care about, all that is important to me at this phase in my life, everyone dances around it- I would give up showers, eating, and work….this is what I care about as a 20-year old man”.

#### Roles and participation

##### Goals

Participants noted that higher function entails *“knowing where you want to go and trying to achieve that.”* Additionally, participants spoke of the importance of *“hope”* or *“vision”* for high function. Beyond setting goals, participants expressed that higher function entails “*following through with plans or goals*,” and that when functioning well, “*plans that are in your head come into fruition and actions are executed fully*.” Participants noted that this was a “*large range”* and *“can be as simple as getting out of bed or working for a year.”* Goals were framed in relation to life transitions and societal roles, with a participant expressing that low function entails not reaching *“what’s expected when you grow up.”* Participants provided examples such as *“dogs, kids, house,*” and *“moving out.”*

##### School/work

Participants mentioned that high function means to *“go to school”* or *“go to work.*” Additionally, participants depicted school and work as key goals, with one participant describing a goal of *“being a social worker.”* Many participants reporting being out of work or school, but highlighting this was critical to high levels of function. However participants also noted many barriers to returning to school/work, including disclosing a mental health challenge, low pay, complications with disability laws, and extended time away from the workforce or education system.

##### Daily life management

Participants expressed that function necessitates “*life skills.”* Examples given were *“to keep reminders”*, *“cooking”* and *“cleaning”.* Additionally, managing finances and medication was also mentioned. Being able to organize time was also brought up by participants, as was *“balancing between needs and wants.”* Managing daily life did not always entail maintaining the status quo, with participants noting that higher function may encompass *“leaving your comfort zone,”* and *“breaking, maybe, a habit that would’ve caused you issues to begin with.”*

##### Service use

Low function was depicted as *“not making it to appointments.”* Services highlighted as important to function include *“psychiatrists,”* and *“case managers, peer support workers, doctors,”* and *“outreach workers.”* Participants also spotlighted that function entails being able to use transit to get to appointments. Participants stressed that often appointments were not coordinated and in many locations across the city. One participant noted *“my entire functioning in my day could often be just me getting to where I am supposed to go to see my worker, my doctor, my pharmacist for methadone, and my friends”.*

##### Engagement and enjoyment

Participants highlighted that when on the higher end of the spectrum of function, they participated in activities for their own engagement and enjoyment including *“being mentally active*” and *“doing things you like to do.”* Examples included hobbies, sports or *“learning, not even specifically from schooling but just from like other people.”*

#### Social connection

##### Healthy relationships

Participants described that to have high function, you *“gotta find the right people.”* Low function was depicted as *“inflicting unfairness towards yourself that impacts others around you,”* while high function entails “f*eeling connected to others.”* Participants described that functional gains involved being *“away from toxic people”* or *“people who want to hurt you,”* highlighting the importance of healthy relationships.

##### Social norms

Participants indicated that when they are functioning well, they *“behave in a socially acceptable manner,”* but during periods of low function, *“social cues and behaviours”* are a struggle. Participants also described the importance of *“fitting in”* and reaching *“societal productivity standards”.*

##### Communication

Several participants noted that function means to *“communicate clearly.”* Participants also noted that higher function necessitates “*taking action and engaging with others”*, as well as *“the ability to resolve conflict”.*

##### Support networks

Participants described *“reaching out”* as key to functional gains. Lower states of function involve being *“isolated”* or feeling “*no one they share their feelings with is there to actually help.”* Participants also described *“not being heard”* by healthcare workers during periods of low function but having *“people who are there to sincerely help you,”* when function highly.

### Item bank development (Phase 2)

Based on Phase 1, our team co-developed 97 items with our youth of researchers including the youth peer research and four additional youth researchers (n = 4). Based on the conceptual model, the item set comprised of items from basic needs (n = 50), roles and responsibilities (n = 30), and social connections (n = 17). Based on a preliminary internal review, our team revised 15 items for clarity, and eliminated 13 for repetition, which resulted 84 (basic needs n = 37, roles and responsibly n = 30, social connections (n = 17). We made a choice to include several items for each category to provide a variety of options to participants in phase 3 to consider and choose.

### Cognitive debriefing (Phase 3)

#### Demographics

Participants were aged 20–24 and identified as men (n = 8), women (n = 3), female and non-binary (n = 1), with a variety of ethnic backgrounds (see Table 1). Nine of the participants had high school diplomas and, of these, three had attended university or another form of education. Participants had a range of mental health diagnoses and seven participants were homeless or precariously housed at the time of the study. The breakdown of other participant characteristics are in Table 1.

**Table 1:** Participate demographics. For those who indicated more than one option, a ratio is shown

Demographic Indicator	Focus Group 1 (n = 8)	Focus Group 2 (n = 4)
*Age*		
20–21	1	2
22	3	2
23–24	4	
*Gender*		
Man	7	1
Woman	1	2
Non-binary		1
*Ethnicity*		
Middle Eastern/North African	1	
Black/African	1	
Indigenous	1	1
White	2	2
Middle Eastern/North African	2	
Indigenous & White/Caucasian		1
No applicable option	1	
*Housing*		
I live in a home/apartment that I rent	2	
I live in a single room occupancy (SRO) hotel		0.5
I live with family or a guardian	1	
I am couch surfing	1.5	
I am homeless	1.5	0.5
Other	2	3

#### Item refinement

Following the cognitive debriefing focus groups, we retained 31 items, eliminated 38 (for being not clear, not relevant to function, or lame), revised 15 (for clarity and lameness) and added 4 new ones. Table 2 describes the qualitative criteria considered for item removal or modification. After this phase, 50 items were deemed ready for next phases in the PROM development process: experts by experience review (including young adults accessing services and health providers) and psychometric testing (see Additional file 2: Table S2 for item list). All items were mapped back to the conceptual model from phase 1 (see Fig. 3) including basic needs (n = 26), roles and responsibilities (n = 16), and social connections (n = 8). Participants also noted in this phase that instructions were clear and there was value in having a response scale that had both a numerical options (e.g., 0, 1, 2, 3, 4) and associated descriptors with each number (e.g., none of the time, some of the time, half of the time, most of the time, all of the time). They also noted the value of “time” as descriptors, as it is something most young people have in common and can understand.

**Table 2:** Qualitative criteria considered in the focus groups when considering to items in the candidate item pool

Criteria	Description	Example(s)
Redundancy	Items were eliminated if participants expressed that the concept was better encapsulated in another item	Participants reported that, *“I talk to my doctor”* was represented by *“healthcare team*,” and did not need to be addressed separately
Variance in interpretation	Items were eliminated for having connotations to participants that weren’t intended by our team or outlined by the conceptual model	Regarding, “*I lead a healthy sex life*,” one participant expressed that the term meant, “f*%$ing a lot in my generation.” Items were also eliminated if there could be varying interpretations as to why the question is being asked. For the item “*I do my taxes,”* one participant noted that if this item would make them think “*are you [the health provider] going to audit us now*?”
Youth friendliness and meaningfulness	Item wording did not appeal to youth	For the item, “*I do the things I want to do*”, beyond issues regarding clarity, a participant expressed that the item was “*lame*” and not meaningful
Clarity of relationship to function	The quality or frequency of the items needed to be modified to more clearly depict function	For the item, “*I participate in meaningful activities*,” a participant noted, “*meaningful to who*?” As such, we qualified this to be “*activities that are meaningful to me*.” Several items were also revised to substitute “*access*” for a more active verb such as “*use*”; for example, “*I use health services when I need to,*” since access may also allude to ability to, while use clearly denotes doing Participants expressed concepts underlying items were conducive to function depending on how and when they occurred; as such, adverbs of frequency were modified in some items to depict a functional relationship. For the item, “*I spend time with my family when I want to and/or need too*,” one participant expressed that interaction should be when “*it’s convenient for both parties*,” and as such, we utilized the participant’s suggestion of, “*I maintain healthy relationships*.” In this instance, meeting mutual expectations was expressed as key to function Meeting expectations was also deemed important in items relating to school and work. One participant highlighted that “*just saying I go to school or I go to work doesn’t capture function very well,*” and that you could still be “*failing your classes miserably*.” For other items, engagement needed to be balanced with other demands and personal values for it to denote function. For example, in regard to, “*I exercise enough each day*,” it was noted that some people “*may be busy and not want to,*” so a participant’s suggestions of “*as much as I want to*,” was used to qualify the item instead
Inclusivity	Items were modified to be more inclusive of those in different life circumstances and cultural backgrounds	In regards to saving money, one participant expressed, “*you …only get enough money to pay rent and buy food, then that’s it and you’re done,”* while in regards to preparing healthy meals a participant noted, *“it’s not something everyone has access to.”* As such, we utilized a participant’s suggestion and qualified these items with *“when I can.”* Participants discussed that not everyone uses transit to get about, so we added the item *“I get to where I need to go.”* Likewise, participants suggested that showering, bathing and brushing your teeth could be consolidated into *“one question,”* of *“I maintain my personal hygiene,”* to include those without access to a shower
Conceptual completeness	Items that may not have been included in the conceptual model, but could be considered	Many participants felt that social connection to both friends and family were important, and that the two items could not be consolidated. As such, we added *“My communication with my friends is healthy,”* as well as the item pertaining to family

## Discussion

Function is integral to well-being and mental health, and as such, a PROM measuring this concept has clinical utility in monitoring progress, guiding treatment and communication, and ultimately, helping bridge a road to health and wellness for young adults [41, 42]. However, as outlined in theories of emerging adulthood, what it means to function during this period of life may be distinct from that of adults. As well, what it means to measure for population epidemiological purposes, may be distinct from measuring the effectiveness of treatment over time for individuals [4, 5, 32]. As such, a PROM developed specifically for young adults in this unique stage of life is warranted [43, 44]. In this study an item bank of 50 items was co-developed and refined in collaboration with young adults.

Integrated youth health is a nascent discipline, with significant potential for growth in the next decade [3, 7]. Involving young adults in measurement research leads to improved quality and range of data that is more relevant to this population’s needs [32, 45, 46]. As such, we sought to develop this PROM with, and not just for, young adults. We achieved this by consulting young adult participants during the first two stages of item development and working alongside a peer researcher. We also ensured that all young people, including those who have barriers to accessing mental health services, could participate if they wished. Beyond representing diverse young adults' perspectives, as highlighted by Couch and Frances [47], including marginalized populations in research enables young people to have a voice, self-advocate, and be empowered. Young people in our study emphasized the importance of the concept being clear, meaningful, and not “not lame!”). Being clear and meaningful is in line with studies that highlight that the function construct should be captured from the perspective of the person: Eklund and Argentzell [48] and Edgelow and Krupa [41] both highlight the importance of function capturing the amount of time spent in varying activities, both those they need to do and want to do. Meanwhile, Wada et al. [49] showed that function was tied to the degree to which time allocated to the activity aligned with personal goals and values. Our study also made our team reflect on the importance of items being relevant, engaging, and current (i.e., not lame!). Participants in our cognitive interviews reflected often on how many times they “*have to fill out boring items that do not mean anything to me*” or have a feeling that “*data gets collected for the government and I never see it*!” Participants stressed that it was not only about having a good measure of function, but a process that can move the function data into practice.

Collectively, our study showed that as the demand increases for health services to be youth- and family-centred, there is opportunity for Canada to build a measurement-based care model that is driven by youth-centred data collection, governance, and mobilization efforts. Ultimately, the benefits of such measurement practices could be improved outcomes and experiences for young adults. However, this can only be tested once there is consensus that person-centred outcome measurement be incorporated into performance measures and payment reforms of all integrated youth health services. Without such an approach, youth, families, and health providers may not recognize the improvements) or potential lack of improvements) of young people in Canada and beyond, and young adults may endure ineffective treatment. As Forney and colleagues [32] explain, this is *“particularly problematic for patients from low income and minority groups who face persistent health disparities. The time in long overdue for the field of mental health to embrace measurement based care and live up to medical testing and treat to target principles applied by other medical specialties”* (p.8).

Developing a measure of function for young adults is timely. Compounded by the drug toxicity crisis and COVID-19 in Canada, emergency department visits, hospitalization and suicide in youth have been on a sharp rise since 2007 [50, 51]. The suicide rate among young adults increased from 10.9 per 100,000 population to 11.8 per 100,000 in 2016 [50]. Additionally, according to the latest data from the Canadian Institute for Health Information, emergency department mental health visits for patients aged 12–24 have increased by 85% since 2007. Across Canada, service providers, organizations and decision-makers have struggled to understand what interventions are required for young people (including marginalized and racialized youth) to thrive and succeed [50]. This new item bank leverages existing work in youth engagement and measurement to understand health systems transformation for young Canadians [4, 5, 14, 52]. In partnership with young people, our research highlights the importance of co-creating outcomes and measures that align with their needs. We anticipate PROMs, that are fit for purpose for their context of use, will inform how health services for young people should be developed, implemented, evaluated, and scaled.

One limitation of this study is that we had a relatively small sample size and only conducted focus groups in one geographical area with young adults. This could limit the transferability of our results since as evident in research by Paul [53] and Bolton and Tang [54], what it means to function is bound not just by age, but also geographical, historical, social and cultural context. We also recognize that it is unlikely that saturation may have occurred in two focus groups alone. This is particularly relevant to items relating to technology, which changes rapidly. Likewise, Romaine [55] suggests that language is shaped by social and cultural factors, beyond age. Variable language interpretation was evident in our study, since some items elicited connotations unintended by our team, including our peer researcher, and needed to be modified. Despite the drawbacks of our small sample size, one strength of this study is that our sample had varied ethnic and educational backgrounds, thereby increasing the variability in perspectives imbued. Steel [33] highlights that major barriers to recruiting minorities include utilizing mainstream media for recruitment rather than local connections and failing to convey the value of the research. Our study attracted a diverse group of participants owing to the communication and networking skills of our peer researcher, who carried out recruitment. Nonetheless, further studies would be warranted to verify the conceptual and linguistic applicability in different countries, age groups (notably youth age 12–18), and cultures.

Another potential limitation of this study is that, for practical purposes, we used focus groups rather than individual interviews for cognitive debriefing. Focus groups have been found to generate fewer ideas that are more homogenous than an equivalent number of individual interviews [56, 57]. However, by running two different focus groups, we were able to compare data between them to increase the variability in the information collected. Furthermore, by administering the workbook prior to discussion commencing, we were able to compare private written feedback with opinions shared in a group context. An advantage of focus groups is that they elicit more critical comments, particularly from disempowered groups, thereby maximizing the potential that issues regarding the clarity and conceptual content of items is explored [58, 59]. Nonetheless, it is possible that we may have uncovered additional issues with the item bank had we conducted individual interviews.

A question that arose during the focus groups is whether we are conceptualizing function from a normative perspective, or whether we are addressing how well someone functions given the resources available to them. Participants expressed concern in both focus groups that young peoples’ level of function is dictated not only by capability, but also by access to the services and resources they need to thrive. For example, participants expressed being capable of cooking or showering, but not having access to the amenities to do so. Based on this feedback, where possible, we modified the items to be more inclusive to those with limited access to resources. This has important implications for the clinical utility of this measure; it is a tool to monitor progress, set goals and facilitate conversation regarding function. However, to be of utility in predicting the level of care someone may require, or their capacity to function in the community, we recommend that the data be integrated with subjective report, contextual factors and observational data to optimize how the PROM is interpreted and used.

## Conclusion

This paper reports the early development of a PROM measuring the full breadth of function in young adults. After completing the fourth stage, 50 items are ready for expert review and psychometric testing. The finished PROM will provide a measure for young adults to self-monitor functional gains, while providing clinicians with a tool to assess, monitor and plan interventions in collaboration with young adults facing functional challenges. This PROM fills a gap for a clinically important tool in integrated youth services. Furthermore, since this tool is being developed in consultation with diverse young adults, we are providing a platform for self-advocacy to a population frequently excluded from research. As such, this study makes a significant contribution to literature regarding function in young adults. This PROM developed both for and alongside young adults, will provide a tool to address functional challenges in this population, to help them on their personal journey towards health and wellness.

## Supplementary Information

**Additional file 1.** **Supplemental Table 1.** Interview schedule for Phase 1.

**Additional file 2.** **Supplemental Table 2.** Final items included to move to Phases 4–6.

## Data Availability

The datasets generated and/or analysed during the current study are not publicly available due to privacy but are available from the corresponding author on reasonable request.

## Abbreviations

ICF: International classification of function
WHO: World Health Organization
GAF: Global assessment of functioning
WHODAS: World Health Organization disability assessment schedule
PSP: Personal and social performance scale
SOFAS: Social and occupational functioning assessment scale
L-FAI: Life functioning assessment scale
PROM’s: Patient report outcome measures
CIHI: Canadian Institute for Health Information
