# Supplementary material for: “It cannot be boring!”: Developing a measure of function for young adults accessing integrated youth services
Source: J Patient Rep Outcomes. 2022 Sep 3;6:92. doi: 10.1186/s41687-022-00491-6 (PMC9440742; doi:10.1186/s41687-022-00491-6)
Supplement: Supplementary file 1 — Additional file 1. Supplemental Table 1. Interview schedule for Phase 1. [file 41687_2022_491_MOESM1_ESM.docx]

**Supplemental Table 1. Interview schedule for Phase 1**

[After consent is obtained]

Welcome to our focus group. We acknowledge with gratitude that our work today takes places on the unceded and ancestral territory of the Coast Salish people.

Our aim today is to explore a concept of function. Why are we developing a new measure of function? Young people at Foundry have told us that function is important to them, but that we only measure mental health challenges and other things that may be going wrong. Young people have told us in other studies that function is important to them. Having a measure that works for young adults in {blinded for review} is critical to guide care and design interventions that meet the needs of young adults. We thank you for your participation today.

On the back wall, you will notice a large arrow drawn on the wall. We are here to think about what function means to you. We are also here to discuss what it means to go from low to high function. We recognize you all have different experiences and some of these discussions may trigger uncomfortable feelings. Our team is here to support you. If you feel that you need to leave for any reason, please check in with {blinded for review}.

Here is the plan for the day. We will be together for 90 minutes. We will be asking you questions about function. We want to create a safe space for everyone to participate. You may write, draw, or contribute by putting up your hand and speaking. Everything we say is confidential and we ask that it stays in this room.

Before we begin, are there any questions?

1. *What does function mean to you?*
2. *What does it look like to go from low to high function?*
3. *What does it look like to go from high to low function?*
4. *What does a person look like who is in the “high function” area?*
5. *What does a person look like who is in the “low function” area?*
6. *What are important factors to keep a person in the high function range?*
7. *What are important factors that may keep a person in the low function range?*

Thank you for participating. This is a process with several steps. We may reach out to ask you for clarification about some questions and to ask you to validate the results of the study. This means that we may ask you if the results reflect the group’s intention and your personal contributions. If you do not wish to participate in any further parts of this study, please connect with the study lead or our youth research lead (blinded for review).
