# Supplementary material for: “It cannot be boring!”: Developing a measure of function for young adults accessing integrated youth services
Source: J Patient Rep Outcomes. 2022 Sep 3;6:92. doi: 10.1186/s41687-022-00491-6 (PMC9440742; doi:10.1186/s41687-022-00491-6)
Supplement: Supplementary file 2 — Additional file 2. Supplemental Table 2. Final items included to move to Phases 4–6. [file 41687_2022_491_MOESM2_ESM.docx]

**Supplemental Table 2.** Final items included to move to Phases 4-6

1. I get enough sleep.
2. I get out of bed in the morning with ease.
3. I ask for help when I need to.
4. I use the supports available to me when I need them.
5. I use positive strategies to cope with stress.
6. I overcome setbacks.
7. I adapt to new habits when facing life challenges.
8. I use healthy strategies to manage pain when I need them.
9. I talk to my healthcare team when I need to.
10. I talk to my service providers when I need to.
11. I use health services when I need to.
12. I take time for myself when I need it.
13. I help people when they need me.
14. I set goals.
15. I meet my goals.
16. I follow my plan to achieve my goals.
17. I have a daily routine that works well for me.
18. I do what is expected of me (eg. school, work, healthcare plan, childcare, etc)
19. I keep up with school and/or work.
20. I eat a balanced diet.
21. I buy groceries when I need to.
22. I prepare healthy meals when I can.
23. I shower/bathe when I need to.
24. I maintain my personal hygiene.
25. I brush my teeth twice a day.
26. I maintain a clean room/house.
27. I keep my personal space clean.
28. I do my laundry when needed.
29. I do my dishes when needed.
30. I maintain healthy relationships.
31. My communication with my family is healthy.
32. My communication with my friends is healthy.
33. I communicate my needs to my friends.
34. I set the personal boundaries I need.
35. I listen to the person I am having a conversation with.
36. I participate in things that I enjoy.
37. I participate in activities that are meaningful to me.
38. I spend money on things I need.
39. I budget my money.
40. I keep track of my expenses.
41. I use bank services as needed.
42. I save money when I can.
43. I get to where I need to go.
44. I use transportation when I need it.
45. I exercise as much as I want to.
46. I practice safe sex.
47. I control my use of social media/technology.
48. I control my use of substances (eg. cigarettes, alcohol, cannabis, opiates, etc).
49. I carry out my cultural practices as much as I want to.
50. I practice my beliefs as much as I want to.
